# The effect of shareholder litigation rights on female board representation: A quasi-natural experiment

**DOI:** 10.1371/journal.pone.0272792

**Published:** 2022-09-21

**Authors:** Pattanaporn Chatjuthamard, Ploypailin Kijkasiwat, Pornsit Jiraporn

**Affiliations:** 1 Sasin Graduate Institute of Business Administration of Chulalongkorn University, Bangkok, Thailand; 2 Khon Kaen University, Khon Kaen, Thailand; 3 School of Graduate Professional Studies, Pennsylvania State University, Malvern, PA, United States of America; University of Almería, SPAIN

## Abstract

Employing as a quasi-natural experiment an unexpected judgment by the Ninth Circuit Court of Appeals that raised the difficulty of shareholder litigation, we explore the effect of shareholder litigation rights on board gender diversity. Our difference-in-difference estimates show that an exogenous reduction in shareholder litigation risk results in significantly less female board representation, a decline by 11.44% in particular. Our findings corroborate the view that strong shareholder litigation rights strengthen internal governance such as board oversight. Therefore, when shareholder litigation rights are weakened, there is a drop in board quality. Additionally, we document a decline in board independence and an increase in board size following the Ninth Circuit ruling, both of which are associated with poorer board monitoring. Further analysis validates the results. i.e., propensity score matching, entropy balancing, GMM dynamic panel data estimation, and Oster’s (2019) testing for coefficient stability. Based on a quasi-natural experiment, our conclusion probably reflects a causal influence, rather than a mere correlation.

## Introduction

Prior research has carefully examined different internal and external governance instruments. The possibility of shareholder litigation is one of the most distinctive external governance mechanisms. Because the threat of shareholder lawsuits deters opportunistic management from exploiting shareholders, shareholder litigation risk functions as a kind of governance. When managers risk possible legal ramifications, they are less likely to take advantage of shareholders, thereby minimizing the agency conflict. In the domains of law, economics, finance, and accounting, there is a considerable body of literature on shareholder litigation risk [1–5]. This is obviously a critical area of research in the literature. Exploiting as a quasi-natural experiment the unexpected ruling of the Ninth Circuit Court of Appeals that raised the difficulty of shareholder litigation, we contribute to the literature by exploring the effect of shareholder litigations rights on board quality with a focus on board gender diversity.

Female representation on corporate boards is vital, not just for academics, but also for investors, shareholders, regulators, and lawmakers. For example, on August 29, 2018, California legislators approved a legislation emphasizing the significance of board gender diversity by forcing large publicly listed companies in the state to have at least one female director by the end of 2019 or face financial penalties. Numerous countries, including Australia, Belgium, France, Germany, Iceland, Italy, Norway, and Spain, have implemented legislations mandating female representation on corporate boards of directors [6, 7]. The academic literature has generated a wealth of research on board gender diversity [8–16]. Without a doubt, this is a vast and important area of investigation in the literature.

One area of research that has been lacking in the literature is the determinants of board gender diversity. While most research in this area focuses on the effect of board gender diversity on various corporate outcomes, very little research explores the factors that influence board gender diversity. One notable exception is a study by Chatjuthamard et al. [17], which examines how the takeover market affects board gender diversity. Otherwise, there is a serious lack of research on how board gender diversity is determined. We address this important gap in the literature by investigating how shareholder litigation rights influence female board representation. Our study is the first to link shareholder litigation rights to board gender diversity.

Employing an unexpected verdict of the Ninth Circuit Court as an exogenous shock, our difference-in-difference estimates demonstrate that an exogenous reduction of shareholder litigation rights results in a significantly lower level of female board representation. Our findings corroborate the complement hypothesis, where shareholder litigation rights and boards of directors function as complementary governance instruments, rather than substitutes. Stronger shareholder litigation rights strengthen board gender diversity and board quality. That is why an exogenous decline in shareholder litigation rights weakens board quality considerably. In particular, an exogenous decline in shareholder litigation rights that can be ascribed to the Ninth Circuit decision brings about an 11.44% reduction in female board representation. Hence, not only is the effect statistically significant, it is also economically palpable. As our identification strategy is based on an exogenous shock from outside the firm, endogeneity is much less likely. Consequently, our results probably reflect a causal influence, rather than just a correlation. At any rate, to ensure that our results are robust, we perform additional robustness checks, i.e., propensity score matching, entropy balancing, GMM dynamic panel data estimation, and Oster’s [18] testing for coefficient stability. The results are validated by all the robustness checks. Our conclusion is unlikely tainted by endogeneity.

It is important to emphasize that our study overcomes a few challenges associated with the research in this area. First, it is difficult to identify an exogenous shock to shareholder litigation rights. By using the unexpected Ninth Circuit judgement, we utilize a shock from outside the firm that is unrelated to firm characteristics. This is crucially important as the literature in this area has been plagued by endogeneity. Our empirical strategy alleviates the endogeneity problem substantially. Second, little research has been conducted on the determinants of board gender diversity. This is a crucial challenge in the literature. We overcome this problem by showing that shareholder litigation rights constitute one of the most important determinants of female board representation.

The findings of our paper aptly extend several vital areas of the literature. First, we contribute to the growing body of knowledge about the effects of shareholder litigation risk on corporate strategies, policies, and outcomes [19–35]. Our findings add to this body of knowledge by revealing that the possibility of shareholder litigation has a significant impact on female board representation. Our analysis is the first to establish a relationship between shareholder litigation rights and board gender diversity.

Second, we contribute to an important field of research on board gender diversity. The vast majority of prior studies in this area have focused on the effect of female board representation on financial performance [8–16, 36–39]. Our research, on the other hand, looks into how shareholder litigation rights affect board gender diversity. Because we find that shareholder litigation rights have a significant influence on board gender diversity, as well as on board size and board independence, our results imply that board gender diversity constitutes a crucial aspect of the board of directors, possibly just as important as board independence and board size. If board gender diversity were unimportant, there would not be a significant impact.

Finally, we contribute to a relatively new but rapidly rising area of research that leverages the Ninth Circuit decision as an exogenous shock. Recent research has examined the effects of litigation risk on a number of corporate outcomes using this empirical approach [5, 40–46]. We contribute significantly to the corpus of knowledge in this area by applying this empirical method to female board representation.

Furthermore, our study offers several key implications with practical value. First, shareholders learn from our study that their litigation rights are crucial. Their ability to file lawsuits represent an important instrument of external governance and has a palpable effect on internal governance mechanisms, such as board characteristics. Second, our results are also useful to regulators and legislators, who design laws and regulations that safeguard shareholders. Our results suggest that any regulation on board governance should take into account the effect of external governance mechanisms, such as shareholder litigation rights. In addition, judges also benefit from our findings as they are informed that their rulings have far and wide implications that influence companies’ internal governance arrangements.

## Pertinent research and hypothesis development

### Board gender diversity

Several theories, including resource dependence theory and agency theory, can explain the relevance of female board representation. Companies, according to the resource dependence theory, seek to attract and hire board members who complement their present resource profile and may offer new types of human and social capital to the firm [47–49]. Furthermore, according to this view, increasing the size and diversity of the board of directors can develop a strong link between firms and their external environment [47, 49, 50]. Female directors contribute to the board distinct insights and experiences. These are significant resources that should help the board perform better as a corporate governance instrument. Firm performance is expected to be stronger as a result of improved board effectiveness, according to this view.

Agency theory addresses the conflicts of interest between principals (e.g., shareholders) and agents (e.g., managers), as well as the function of the corporate board in monitoring and resolving these conflicts [51, 52]. Diversity, according to agency theory, improves the board of directors’ monitoring responsibility. Carter, Simkins, and Simpson [11], Adams and Ferreira [8], and Adams, Nowland, and Grey [53], for example, employ agency theory to examine the link between gender diversity on the board and company value and find a positive association between gender diversity and firm performance. According to Adams and Ferreira [8] and Adams et al. [53], female directors have stronger monitoring capacities since they think independently, and board gender diversity strengthens management responsibility by increasing board meeting attendance and CEO accountability [49]. The increase in board monitoring that may be attributed to board gender diversity improves business performance.

There are also some arguments against board gender diversity. Organizations may select boards with gender diversity for the sake of optics, yet they may not adequately use people’s diverse contributions [54, 55]. Furthermore, some studies imply that gender diversity in boardrooms may lead to board disagreements, harm board cohesion, and, as a result, harm business performance and competitive advantage [56–59]. Additionally, women may not be given the power they require to make proper decisions on some boards, which may result in poor performance [60].

The empirical evidence regarding the effect of board gender diversity on business performance is inconclusive [7]. Numerous studies show positive relationships [9–16], while others establish no link [8, 38, 39]. Due to the ambiguity of the empirical evidence, the controversy over the costs and advantages of gender diversity on corporate boards of directors continues. Instead of focusing on the effect of board gender diversity on firm performance, our study contributes to the debate on this issue by investigating the effect of shareholder litigation rights on female board representation. As far as we are aware, our study is the first to do so in the literature.

### Shareholder litigation and corporate governance

Legal safeguards protecting shareholders’ interests are commonly recognized as an essential component of corporate governance. Stockholders, in particular, have the right to sue management if they think the executives are abusing their positions of authority [61]. Indeed, a large corpus of research exists on the motivations and results of shareholder lawsuits [62, 63]. Potential shareholder lawsuits significantly raise the cost of management’s opportunistic actions and serve as a considerable disincentive for such managers. As a result, litigation risk is seen as a critical external corporate governance instrument [40, 64]. Shareholder litigation, on the other hand, may be controversial. Many individuals argue that the vast majority of class action lawsuits are frivolous and that attorneys make a fortune at the expense of shareholders [5, 34, 65].

The literature has examined the role of shareholder litigation as an external governance mechanism. Shareholder litigation, according to Niehaus and Roth [66], increases CEO turnover rates, with the impact being strongly related to the merits of the action. While independent directors do not experience noticeable turnover, they do see a significant decline in the number of future board seats they hold, showing that shareholder litigation has some disciplinary effect on independent directors, according to Fich and Shivdasani [67], Chu [5] Jaroenjitrkam, Treepongkaruna, and Jiraporn [34].

### Hypothesis development

Based on the literature, two hypotheses can be advanced. First, the substitution hypothesis argues that, as shareholder litigation rights are weakened, the quality of board monitoring rises to compensate. Because litigation risk functions as an external governance mechanism, when it is weakened, an internal governance mechanism, i.e., the board of directors becomes stronger as a result. There is a substitution effect. Greater board gender diversity has been argued to be beneficial, according to the resource dependence theory and agency theory. There is also ample empirical evidence in favor of the beneficial role of female directors. Therefore, this view suggests that an exogenous drop in shareholder litigation risk results in greater female board representation. This is the substitution hypothesis.

On the contrary, two governance mechanisms may be complements, rather than substitutes. Shareholder litigation rights, acting as an external governance instrument, prevent self-interested and opportunistic managers from subverting other governance mechanisms inside the firm, resulting in more effective internal governance. When shareholder litigation rights are compromised, managers are better able to take actions that do not enhance shareholder value, such as weakening the monitoring function of the board of directors. Consequently, this view argues that an exogenous decline in shareholder litigation risk brings about lower board gender diversity. This is the complement hypothesis.

### The Ninth Circuit verdict as a quasi-natural experiment

The Private Securities Lawsuits Reform Act (PSLRA) was passed by Congress in December 1995 as part of a larger movement to protect companies against fraudulent shareholder lawsuits. Prior to the PSLRA, plaintiffs may claim that a steep decline in stock prices showed that the issuer and its management suppressed unfavorable information that caused the drop. In complaints alleging fraud, the PSLRA requires particular evidence to create a compelling inference that the defendant acted with the requisite state of mind. However, various federal circuit courts in the United States interpret the legal pleading requirements differently. Plaintiffs must show that the defendants were willfully negligent in creating the falsehood that gave rise to the fraud accusation, according to the Ninth Circuit [5]. The United States courts of appeals, sometimes known as circuit courts, are the intermediate appellate courts of the United States’ federal judiciary. The courts are divided into 13 circuits, each of which hears appeals from district courts within its jurisdiction. Following the Supreme Court, the courts of appeals in the United States are recognized as the most powerful and influential courts in the country (Jaroenjitrkam, Treepongkaruna, and Jiraporn, 2021).

The July 2, 1999, court decision had a disproportionate impact on corporations located in the Ninth Circuit. Due to the geographical dispersion of stockholders, securities class action lawsuits can be filed in any of the federal circuit courts. Consequently, the ruling of the Ninth Circuit Court is expected to have a stronger impact on firms having headquarters in the Ninth Circuit. Indeed, Johnson et al. [65] find that corporations headquartered in the Ninth Circuit generate much higher judgment announcement returns than other firms. Pritchard and Sale [68] report that the Ninth Circuit dismisses class action lawsuits far more frequently than other circuits following the verdict.

Several recent studies use the Ninth Circuit verdict as an exogenous shock to examine the impact of shareholder litigation risk on corporate strategies and outcomes. Chu [5], for instance, shows that increasing the difficulty of shareholder litigation decreases loan spreads considerably, supporting the idea that shareholder litigation allows shareholders to recover value from creditors following the declaration of a bankruptcy. Huang, Roychowdhury, and Sletten [43] and Liao and Ouyang [40] report that, when corporations are more protected against shareholder lawsuits, they exhibit greater real earnings management. Arena, Wang, and Yang [44] document a rise in tax avoidance as a result of the Ninth Circuit decision, implying that the possibility of litigation works as a disincentive against tax evasion.

Jaroenjitrkam, Treepongkaruna, and Jiraporn [34], exploiting the Ninth Circuit decision as a quasi-natural experiment, show that when shareholder litigation risk is reduced, companies become much more socially responsible. They suggest that when litigation risk is lowered, managers have greater incentives to support long-term investments in corporate social responsibility (CSR). Although this study relies on the same identification strategy as ours, the focus of their study is on CSR, whereas the focus of our study is on corporate governance and board characteristics. Therefore, while our studies share a similar approach, they are distinct from each other. Numerous further recent studies use the Ninth Circuit judgment as quasi-natural experiment [40, 42–45].

## Sample selection, data description, and empirical strategy

### Sample selection

We analyze a large sample of U.S. firms. The data on directors and board characteristics are from the Institutional Shareholder Services (ISS). Firm-specific characteristics are from COMPUSTAT. Outliers are winsorized at the 1% and 99% levels respectively. The final sample contains 19,631 firm-year observations from 1996 to 2016. Our sample period starts in 1996 because the data for board characteristics are available beginning in 1996. Our primary variable of interest is board gender diversity, which is defined as the percentage of female directors on the board, consistent with the literature in this area.

### Empirical strategy

Following the literature in this field, we perform a difference-in-difference analysis. There are two binary variables generated. First, Ninth Circuit is a binary variable that equals one if the company’s headquarters is in the Ninth Circuit and zero otherwise. Alaska, Arizona, California, Hawaii, Idaho, Montana, Nevada, Oregon, and Washington are all included in the Ninth Circuit Court of Appeals. Second, Post-1999 is a binary variable with a value of one after 1999 and a value of zero otherwise. In addition, an interaction term is constructed between the two binary variables. The difference in differences is denoted by the coefficient of the interaction variable. Essentially, the following regression analysis is estimated.

$$\%\;Female\;Directors_{it}=\;\alpha \;+\;\beta_{1}{\left(Ninth\;Circuit\right)}_{i}+\;\beta_{2}{\left(Post-1999\right)}_{t}+\;\beta_{3}{\left(Ninth\;Circuit\;\times \;Post-1999\right)}_{it}+\;\beta_{4}{\left(Controls\right)}_{it}$$

where i indexes firms and t indexes years.

Because we control for firm fixed effects, Ninth Circuit is dropped. Similarly, Post-1999 is subsumed by year fixed effects. Furthermore, we include several control variables that might affect board gender diversity Specifically, we include profitability (earnings before interest and taxes [EBIT]/total assets), firm size (natural log of total assets), leverage (total debt/total assets), capital investments (capital expenditures/total assets), intangible assets (advertising and research and development [R&D] expenses/total assets), cash holdings (cash holdings/total assets), dividend payouts (dividends/total assets), and discretionary spending (selling, general, and administrative expenses [SG&A]/total assets). The S1 Appendix displays the variable definitions. Table 1 shows the descriptive statistics for all the variables.

**Table 1:** Summary statistics. Board gender diversity is the percentage of female directors on the board. Board independence is the percentage of independent directors on the board. Board size is the number of directors. SG&A is selling, administrative, and general expense.

	Mean	S.D.	0.25	Median	0.75
% Female Directors	10.781	9.817	0.000	11.111	16.667
% Independent Directors	71.589	16.704	62.500	75.000	85.714
Board Size	9.150	2.316	7.000	9.000	11.000
Total Assets	7824.198	28000.000	657.440	1723.402	5294.216
Total Debt/Total Assets	0.227	0.174	0.074	0.223	0.344
EBIT/Total Assets	0.099	0.084	0.058	0.095	0.142
Capital Expenditures/Total Assets	0.056	0.051	0.022	0.040	0.071
Advertising Expense/Total Assets	0.012	0.028	0.000	0.000	0.008
R&D Expense/Total Assets	0.027	0.047	0.000	0.000	0.034
Dividends/Total Assets	0.013	0.019	0.000	0.006	0.020
Cash Holdings/Total Assets	0.140	0.159	0.023	0.077	0.204
SG&A Expense/Total Assets	0.217	0.189	0.071	0.177	0.316

## Results

### Baseline regression analysis

The difference-in-difference estimates are shown in Table 2 Panel A. We do not include the control variables in these regressions. To the extent that some of the control variables are endogenous, the results might be biased [69]. To avoid this possibility, we start without including any control variables. In Model 1 where the dependent variable is the percentage of female directors, the coefficient of the interaction term is significantly negative. Thus, an exogenous reduction in shareholder litigation rights that can be ascribed to the Ninth Circuit judgment reduces board gender diversity significantly.

**Table 2:** The effect of shareholder litigation rights on board quality. Ninth Circuit is a binary variable equal to one if the company is located in the jurisdiction of the Ninth Circuit. Post-1999 is equal to one after 1999 and zero otherwise. Board gender diversity is the percentage of female directors on the board. SG&A is selling, administrative, and general expense. The rest of the variable definitions are shown in the S1 Appendix.

**Panel A: Excluding firm-specific variables.**			
	(1)	(2)	(3)
	% Female Directors	% Independent Directors	Ln (Board Size)
**Ninth Circuit × Post-1999**	-1.123**	-1.330**	0.055***
	**(-2.225)**	**(-2.526)**	**(3.766)**
Constant	11.051***	71.931***	2.284***
	(127.645)	(796.233)	(912.359)
Firm Fixed Effects	Yes	Yes	Yes
Year Fixed Effects	Yes	Yes	Yes
Observations	19,323	19,323	19,323
Adjusted R-squared	0.708	0.694	0.754
**Panel B: Including firm-specific characteristics**			
	(1)	(2)	(3)
	% Female Directors	% Independent Directors	Ln (Board Size)
**Ninth Circuit × Post-1999**	-1.347**	-1.534***	0.039***
	**(-2.649)**	**(-3.015)**	**(3.263)**
Firm Size	0.689**	0.906	0.080***
	(2.354)	(1.513)	(15.977)
Leverage	-0.368	-0.704	0.005
	(-0.353)	(-0.505)	(0.195)
Profitability	-0.662	-1.790	-0.032
	(-0.477)	(-0.785)	(-1.276)
Capital Investments	-3.544**	-1.401	-0.097
	(-2.352)	(-0.385)	(-1.464)
Advertising Intensity	-16.597**	-16.201	-0.119
	(-2.336)	(-1.340)	(-0.921)
R&D Intensity	-9.539*	-7.078	0.099
	(-1.949)	(-0.793)	(1.539)
Dividends	15.576***	10.611	0.549***
	(3.294)	(0.982)	(5.149)
Cash Holdings	-0.074	4.353***	-0.039*
	(-0.089)	(3.737)	(-1.738)
Discretionary Spending	4.138***	6.380*	0.142***
	(3.833)	(1.947)	(6.354)
Constant	5.551**	63.732***	1.653***
	(2.443)	(12.258)	(41.229)
Firm Fixed Effects	Yes	Yes	Yes
Year Fixed Effects	Yes	Yes	Yes
Observations	19,323	19,323	19,323
Adjusted R-squared	0.709	0.695	0.768

Robust t-statistics in parentheses

*** p<0.01,

** p<0.05,

* p<0.1

Because prior research generally finds that female board representation is beneficial, a decline in board gender diversity is viewed as a disadvantage that lowers the quality of board monitoring. To verify that this is the case, we explore two board attributes that have been widely recognized in the literature, i.e., board size and board independence. There is considerable evidence in the literature that greater board independence and smaller board size are advantageous [70–74]. Model 2 and Model 3 have board independence and board size as the dependent variables respectively. The coefficients of the interaction term are significantly negative in Model 2 and significantly positive in Model 3. Therefore, a drop in litigation risk results in significantly lower board independence and significantly larger board size, suggesting a decline in board quality. The findings confirm the advantage of female board representation, which is reduced when shareholder litigation rights are weakened. To further validate our findings, we add the control variables in Table 2 Panel B. The results remain consistent.

To estimate the economic significance, we make the following calculations. The coefficient of the interaction term in Model 1 is -1.123, implying that an exogenous drop in litigation risk attributable to the Ninth Circuit ruling causes the percentage of female directors to drop by 1.123%. Because the standard deviation of the proportion of female directors is 9.817, a decline by 1.123 represents an 11.44% reduction in female board representation. Therefore, not only is the effect statistically significant, but it is also economically meaningful.

### Propensity score matching

While our identification strategy is already significantly less susceptible to endogeneity, we use propensity score matching to further validate our findings Rosenbaum and Rubin [75], Lennox, Francis, and Wang [76], Ongsakul, Chatjuthamard, Jiraporn, and Chaivisuttangun [77], Chatjuthamard, Jiraporn, and Treepongkaruna [78], Padunsaksawasdi, Treepongkaruna, Jiraporn, and Uyar [79], Chatjuthamard, Jiraporn, and Lee [80]. Following the judgment, those corporations in the Ninth Circuit are considered the treatment group. We choose a firm outside the treatment group that is most similar to each company in the treatment group using nine company-specific attributes (i.e., the nine control variables in the regression analysis). Our treatment and control firms are therefore almost identical in every observable characteristic, with the exception of the degree of shareholder litigation risk.

We conduct diagnostic testing to ensure the accuracy of our matching. The results are displayed in Table 3 Panel A. Model 1 is a logistic regression with a binary dependent variable equal to one if the company is in the treatment group and zero otherwise. Model 1 includes the whole sample (pre-match). The regression result indicates that the treatment firms are significantly different from the rest of the sample in a number of ways. Specifically, the treatment firms are larger in size, make less capital investments, spend more on advertising, invest more in R&D, and pay smaller dividends, and hold more cash. These material differences should be accounted for to ensure that our results are not biased.

**Table 3:** Propensity score matching. Ninth Circuit is a binary variable equal to one if the company is located in the jurisdiction of the Ninth Circuit. Post-1999 is equal to one after 1999 and zero otherwise. Board gender diversity is the percentage of female directors on the board. SG&A is selling, administrative, and general expense. The rest of the variable definitions are shown in the S1 Appendix.

**Panel A: Diagnostic testing**			
	(1)	(2)	
	Pre-Match	Post-Match	
	Treatment	Treatment	
Firm Size	0.087*	0.074	
	(1.865)	(1.529)	
Leverage	-0.548	-0.008	
	(-1.319)	(-0.021)	
Profitability	-0.339	-0.460	
	(-0.620)	(-0.821)	
Capital Investments	-3.103**	-0.393	
	(-2.400)	(-0.299)	
Advertising Intensity	6.805***	0.127	
	(2.956)	(0.058)	
R&D Intensity	7.191***	1.263	
	(5.609)	(1.016)	
Dividends	-9.117***	-0.699	
	(-2.584)	(-0.212)	
Cash Holdings	2.785***	-0.215	
	(7.894)	(-0.610)	
Discretionary Spending	-0.640	-0.198	
	(-1.460)	(-0.448)	
Constant	-2.498***	-0.440	
	(-6.034)	(-1.026)	
Pseudo R-squared	0.105	0.004	
Observations	19,631	6,698	
**Panel B: The effect of shareholder litigation rights on board gender diversity**			
			(1)
			% Female Directors
**Ninth Circuit × Post-1999**			-1.112*
			**(-1.802)**
Firm Size			1.417***
			(4.811)
Leverage			-2.593
			(-1.417)
Profitability			0.949
			(0.576)
Capital Investments			-4.677
			(-1.114)
Advertising Intensity			-11.333*
			(-1.818)
R&D Intensity			-1.776
			(-0.268)
Dividends			23.932**
			(2.564)
Cash Holdings			0.523
			(0.515)
Discretionary Spending			4.695**
			(2.295)
Constant			-0.413
			(-0.163)
Firm Fixed Effects			Yes
Year Fixed Effects			Yes
Observations			6,206
Adjusted R-squared			0.733

Robust t-statistics in parentheses

*** p<0.01,

** p<0.05,

* p<0.1

Model 2 is a logistic regression for the propensity-score matched sample (post-match). None of the coefficients in Model 2 are significant. As a result, our treatment and control firms are statistically similar in all observable aspects. To the extent that shareholder litigation risk is irrelevant, our treatment and control firms should also be comparable in terms of female board representation. The regression result for the PSM sample is shown in Table 4. The interaction term has a negative and significant coefficient, implying that female board representation decreases significantly. Due to the consistency of our PSM results, it is improbable that our conclusion is contaminated by endogeneity.

**Table 4:** Entropy balancing. Ninth Circuit is a binary variable equal to one if the company is located in the jurisdiction of the Ninth Circuit. Post-1999 is equal to one after 1999 and zero otherwise. Board gender diversity is the percentage of female directors on the board. SG&A is selling, administrative, and general expense. The rest of the variable definitions are shown in the S1 Appendix.

	(1)
	% Female Directors
**Ninth Circuit × Post-1999**	-0.930***
	**(-2.749)**
Firm Size	1.238***
	(4.322)
Leverage	-0.920
	(-0.466)
Profitability	0.975
	(0.795)
Capital Investments	-6.103**
	(-2.646)
Advertising Intensity	-12.067**
	(-2.178)
R&D Intensity	-0.833
	(-0.229)
Dividends	18.286***
	(3.409)
Cash Holdings	-0.243
	(-0.288)
Discretionary Spending	3.304**
	(2.472)
Constant	1.122
	(0.499)
Firm Fixed Effects	Yes
Year Fixed Effects	Yes
Observations	19,323
Adjusted R-squared	0.718

Robust t-statistics in parentheses

*** p<0.01,

** p<0.05,

* p<0.1

### Entropy balancing

Consistent estimates and identification of the treatment effect in the presence of conditional mean independence require that the variables used to estimate the average treatment effect be balanced, i.e., they have a comparable statistical distribution. To address this issue, we employ the entropy balancing approach developed by Hainmueller and Xu [81]. Entropy balancing enables users to fit weights that fulfill a large variety of possible balance criteria, including accurate balance on the treatment’s moments and reweighted control group covariate distributions [82]. We require that the covariates’ first, second, and third moments are balanced. This unique approach of matching has been extensively employed in recent publications (Chatjuthamard, Jiraporn, and Lee, 2021). Table 4 summarizes the regression result following entropy balancing. The interaction variable’s coefficient remains negative and significant. Due to an unanticipated drop in shareholder litigation risk, female board representation is weakened.

### GMM dynamic panel data analysis

To further corroborate the findings, we use a dynamic GMM panel estimator to examine the influence of shareholder litigation rights on board gender diversity. This strategy makes use of the dynamic relationships that exist between the explanatory variables. To avoid any possible bias caused by time-invariant unobserved heterogeneity, the variables are first differenced. Following first-differencing, we employ GMM to assess the influence of litigation risk on female board representation, utilizing lagged values of the explanatory variables as instruments for the current explanatory variables. This method is far less susceptible to the omitted-variable bias. The result is shown in Table 5. The coefficient of the interaction term remains negative and significant, again confirming a significant decline in board gender diversity following the Ninth Circuit judgment.

**Table 5:** GMM dynamic panel data analysis. Ninth Circuit is a binary variable equal to one if the company is located in the jurisdiction of the Ninth Circuit. Post-1999 is equal to one after 1999 and zero otherwise. Board gender diversity is the percentage of female directors on the board. SG&A is selling, administrative, and general expense. The rest of the variable definitions are shown in the S1 Appendix.

	(1)
	% Female Directors
**Ninth Circuit × Post-1999**	-27.718***
	**(-3.847)**
Firm Size	1.368***
	(3.915)
Leverage	0.082
	(0.099)
Profitability	-1.346
	(-1.074)
Capital Investments	-2.666
	(-1.175)
Advertising Intensity	-17.452**
	(-2.072)
R&D Intensity	-0.091
	(-0.017)
Dividends	3.339
	(0.614)
Cash Holdings	2.330**
	(2.484)
Discretionary Spending	4.107**
	(2.426)
% Female Directors (t-1)	0.502***
	(19.022)
% Female Directors (t-2)	0.019
	(1.523)
Constant	-5.813**
	(-2.298)
Year Fixed Effects	Yes
Observations	10,675

z-statistics in parentheses

*** p<0.01,

** p<0.05,

* p<0.1

### Oster’s (2019) testing of coefficient stability

Additionally, to further ensure that our results are not skewed by the omitted-variable bias, we leverage Oster’s [18] insight and estimate the size of the unobservables’ influence necessary to overcome the effect of the observables, thereby reducing the validity of our conclusions. Using Oster’s [18] technique, we calculate that the influence of the unobservables would have to be more than 1.43 times that of the observables in order for our results to be invalidated. In the literature, if the ratio is more than one, the results are considered robust. As a consequence, our findings do not appear to be significantly influenced by the omitted-variable bias.

### Additional robustness checks

To ensure that our results are robust, we execute a couple of additional robustness checks. Table 6 shows the results. First, it could be argued that our entire sample period is too broad. Because the exogenous shock was in 1999, it might be more appropriate to examine a narrower period around the shock. As a result, we run a regression analysis including only the period from 1996 to 2003. In Model 1, the coefficient of the interaction term is significantly negative, again implying a significant drop in board gender diversity. Second, it may be suggested that right after the Nine Circuit decision, the effect of the ruling may not have been so clear. So, we run a regression excluding the transition period, which is 1999 and 2000. The result in Model 2 demonstrates that the coefficient of the interaction is once again negative and significant.

**Table 6:** Robustness checks. Ninth Circuit is a binary variable equal to one if the company is located in the jurisdiction of the Ninth Circuit. Post-1999 is equal to one after 1999 and zero otherwise. Board gender diversity is the percentage of female directors on the board. SG&A is selling, administrative, and general expense. The rest of the variable definitions are shown in the S1 Appendix.

	(1)	(2)
	Shorter Period	Excluding the transition period
	% Female Directors	% Female Directors
**Ninth Circuit × Post-1999**	-1.006***	-1.260**
	(-2.981)	(-2.170)
Firm Size	0.329	0.703**
	(1.177)	(2.355)
Leverage	0.073	-0.720
	(0.091)	(-0.666)
Profitability	-0.621	-0.641
	(-0.397)	(-0.425)
Capital Investments	-3.717*	-4.358**
	(-1.712)	(-2.623)
Advertising Intensity	-8.575	-14.474**
	(-1.083)	(-2.125)
R&D Intensity	-5.266	-11.444**
	(-1.061)	(-2.051)
Dividends	18.096	14.802***
	(1.152)	(3.184)
Cash Holdings	1.066	-0.126
	(1.115)	(-0.138)
Discretionary Spending	3.241***	4.450***
	(3.616)	(4.259)
Constant	5.408**	5.812**
	(2.366)	(2.528)
Firm Fixed Effects	Yes	Yes
Year Fixed Effects	Yes	Yes
Observations	7,026	17,436
Adjusted R-squared	0.759	0.712

Robust t-statistics in parentheses

*** p<0.01,

** p<0.05,

* p<0.1

### Difference in board gender diversity before the Ninth Circuit decision

Our empirical strategy hinges on the parallel trend assumption. It is assumed that the treatment and the control groups would have followed similar trends absent the exogenous shock. This assumption cannot be directly verified because we do not know the counter-factual, i.e., how the treatment firms would have behaved without the Ninth Circuit judgment. This is not observable. What can be done, however, is to make sure that the treatment and the control groups follow similar trends before the shock- a pre-trend analysis. To the extent that there is no difference in board gender diversity before the exogenous shock, any difference that arises later can likely be attributed to the shock.

First, we execute a univariate analysis where we compare board gender diversity between the treatment group, i.e., those in the Ninth Circuit, and the control group, i.e., those outside the Ninth Circuit before 1999. The result is shown in Table 7 Panel A. The difference in the percentage of female directors between the two groups is not statistically significant. So, board gender diversity is similar before the shock. It may be suggested, however, that other additional factors should be considered when making a comparison. So, we perform a regression analysis in Table 7 Panel B. The coefficient of Ninth Circuit is not statistically significant. Again, there is no significant difference in female board representation before the shock. Therefore, the difference in board gender diversity after the Ninth Circuit ruling can probably be ascribed to the shock brought about by the judgment. The effect is thus likely causal, rather than just a correlation.

**Table 7:** Difference in board gender diversity before the Ninth Circuit judgment. Ninth Circuit is a binary variable equal to one if the company is located in the jurisdiction of the Ninth Circuit. Post-1999 is equal to one after 1999 and zero otherwise. Board gender diversity is the percentage of female directors on the board. SG&A is selling, administrative, and general expense. The rest of the variable definitions are shown in the S1 Appendix.

**Panel A: Univariate comparison**						
	Ninth Circuit	Others		Difference	t-statistics	p-value
% Female Directors	6.632	7.177		0.545	1.367	0.172
**Panel B: Multivariate analysis**						
			(1)			
			% Female Directors			
**Ninth Circuit × Post-1999**			**0.824**			
			**(1.524)**			
Firm Size			1.595***			
			(14.094)			
Leverage			0.767			
			(0.724)			
Profitability			0.171			
			(0.078)			
Capital Investments			-4.754*			
			(-1.696)			
Advertising Intensity			2.241			
			(0.245)			
R&D Intensity			-6.018			
			(-1.077)			
Dividends			67.924***			
			(5.735)			
Cash Holdings			0.995			
			(0.719)			
Discretionary Spending			5.186**			
			(2.435)			
Constant			-6.437***			
			(-5.818)			
Firm Fixed Effects			Yes			
Year Fixed Effects			Yes			
Observations			2,638			
Adjusted R-squared			0.132			

Robust t-statistics in parentheses

*** p<0.01,

** p<0.05,

* p<0.1

## Conclusions

One of the most unique external governance instruments is the prospect of shareholder litigation. As the possibility of shareholder litigation deters opportunistic management from exploiting shareholders, shareholder litigation risk operates as a governance mechanism. While prior research has examined the effects of shareholder litigation rights on a plethora of corporate strategies and outcomes, the effect of litigation risk on internal corporate governance has not been investigated. This crucial void in the literature is addressed in this paper, where we explore the influence of shareholder litigation risk on the board of directors, which is widely regarded as the ultimate instrument of internal governance.

Leveraging an unanticipated ruling by the Ninth Circuit Court of Appeals as a quasi-natural experiment, we execute a difference-in-difference analysis and find that an exogenous reduction in shareholder litigation rights leads to significantly less female board representation. As board gender diversity has previously been found to be advantageous, a reduction in board gender diversity implies a decline in board quality. We confirm this argument by showing that an exogenous drop in litigation risk also results in lower board independence and larger board size, which are associated with weaker board oversight. The findings thus corroborate the view that shareholder litigation rights and internal board governance function as complements, rather than substitutes. Stronger shareholder litigation rights improve board gender diversity and board quality. Consequently, when shareholder litigation risk declines, there is a significant reduction in board quality. As our identification strategy is based on a quasi-natural experiment utilizing an exogenous shock, our conclusion is much more likely to demonstrate causality, rather than a mere association.

For robustness, we execute several additional analyses, i.e., propensity score matching, entropy balancing, GMM dynamic panel data analysis, and Oster’s [18] testing of coefficient stability. The conclusion is strongly corroborated by all the robustness checks, further confirming that endogeneity does not pose a serious problem in our study. It is also important to note that our study complements and extends recent studies in this area. For instance, we extend Chatjuthamard, Jiraporn, Lee, Uyar, and Kilic [17]. Their study concentrates on the effect of the takeover market on board gender diversity, regarding the takeover market as an external governance mechanism. Our study is similar in that we also investigate an external governance mechanism that influences female board representation. However, the specific mechanism we examine in our study is shareholder litigation rights, not the takeover market. Therefore, our research is related to theirs, but it is distinct as shareholder litigation is separate from the takeover market. Also, unlike their research, we rely on a quasi-natural experiment, which is more likely to reveal a causal effect, rather than merely an association.

Furthermore, we extend Chatjuthamard, Ongsakul, and Jiraporn [77]. Their research looks at the effect of the takeover market on corporate complexity. Like their research, our study also investigates an important external governance mechanism. Nevertheless, our focus is on shareholder litigation rights, rather than the market for corporate control. Additionally, the outcome investigated in Chatjuthamard, Ongsakul, and Jiraporn [77] is corporate complexity. However, the outcome in our research is female board representation. Therefore, we extend their research by using a distinct external governance mechanism as well as examining a different corporate outcome.

Our empirical findings offer several important practical implications. First, corporate governance experts and shareholder activists should find our results useful as they promote gender diversity on the board and now realize that shareholder litigation rights represent one of the most crucial determinants of female board representation. Likewise, our findings should be interesting to regulators who may be contemplating new regulation requiring gender quotas on the board. The legal community and investors in general also learn from our findings that litigation risk is important and exerts a strong influence on internal corporate governance such as board characteristics.

Our study can be extended in a few ways. First, in addition to board gender diversity, future research can explore the effects of shareholder litigation rights on other aspects of board governance. Second, more effort can be made to identify how the effect of shareholder litigation rights on board gender diversity is different across subgroups. Perhaps, for certain types of firms, the effect is more pronounced. Third, in this study, we rely on the ruling of the Ninth Circuit Court as an exogenous shock, it should be possible for other researchers to exploit a different shock to shareholder litigation rights. For instance, there is a strand of the literature that utilizes universal demand laws as an exogenous shock. Our work can be extended by looking at the impact of the passage of universal demand laws on board characteristics.

## Supporting information

S1 Appendix (DOCX)
