# Supplementary material for: The effect of shareholder litigation rights on female board representation: A quasi-natural experiment
Source: PLoS One. 2022 Sep 21;17(9):e0272792. doi: 10.1371/journal.pone.0272792 (PMC9491617; doi:10.1371/journal.pone.0272792)
Supplement: S1 Appendix — (DOCX) [file pone.0272792.s001.docx]

**Appendix**

**Table A1: Variable definitions**

| **Variable** | **Definition** |
| --- | --- |
|  |  |
| **Ninth Circuit Court** |  |
| Ninth Circuit | This binary variable is equal to one if the firm is located |
|  | the Ninth Circuit Court of Appeals and zero otherwise |
| Post-1999 | This binary variable is equal to one if the year is after 1999 |
|  | zero otherwise. |
| **Board Attributes** |  |
| Board Gender Diversity | % Female Directors |
| Board Independence | % Independent Directors |
| Board Size | Ln (Board Size) |
| **Firm-specific Characteristics** |  |
| Firm Size | Ln (Total Assets) |
| Leverage | Total Debt/Total Assets |
| Profitability | EBIT/Total Assets |
| Capital Investments | Capital Expenditures/Total Assets |
| Advertising Intensity | Advertising Expense/Total Assets |
| R&D Intensity | R&D Expense/Total Assets |
| Cash Holdings | Cash Holdings/Total Assets |
| Dividend Payouts | Dividends/Total Assets |
| Discretionary spending | SG&A Expense/Total Assets |
